# Prevalence and determinants of cessation of exclusive breastfeeding in the early postnatal period in Sydney, Australia

**DOI:** 10.1186/s13006-017-0110-4

**Published:** 2017-04-08

**Authors:** Felix A. Ogbo, John Eastwood, Andrew Page, Amit Arora, Anne McKenzie, Bin Jalaludin, Elaine Tennant, Erin Miller, Jane Kohlhoff, Justine Noble, Karina Chaves, Jennifer M. Jones, John Smoleniec, Paul Chay, Bronwyn Smith, Ju-Lee Oei, Kate Short, Laura Collie, Lynn Kemp, Shanti Raman, Sue Woolfenden, Trish Clark, Victoria Blight, Valsamma Eapen, for the Early Years Research Group, Anne Dudley, Elizabeth Paz, Jacqueline Stack, Karen Sorensen, Mary Knopp, Alison Colley, Carissa Kleiman

**Affiliations:** 1 Centre for Health Research, School of Medicine, Western Sydney University, Campbelltown Campus, Penrith, NSW Australia; 2 Ingham Institute for Applied Medical Research, Liverpool, NSW Australia; 3 School of Women’s and Children’s Health, The University of New South Wales, Kensington, Sydney, NSW Australia; 4 Menzies Centre for Health Policy, Charles Perkins Centre, School of Public Health, Sydney University, Sydney, NSW Australia; 5 School of Public Health, Griffith University, Gold Coast, QLD Australia; 6 Department of Community Paediatrics, Sydney Local Health District, Croydon Community Health Centre, Croydon, NSW Australia; 7 Sydney Children’s Hospital Network, Randwick, NSW Australia; 8 School of Science and Health, Western Sydney University, Campbelltown Campus, Penrith, NSW Australia; 9 Child and Family Health Nursing, Primary & Community Health, South Western Sydney Local Health District, Narellan, NSW Australia; 10 Healthy People and Places Unit, South Western Sydney Local Health, Liverpool, NSW Australia; 11 School of Psychiatry, UNSW Medicine, University of New South Wales, Randwick, NSW Australia; 12 Department of Newborn Care, Royal Hospital for Women, Randwick, NSW Australia; 13 Translational Research and Social Innovation, School of Nursing and Midwifery, Western Sydney University, Penrith, NSW Australia; 14 Community Paediatrician for Child Protection, South Western Sydney Local Health District, Liverpool, NSW Australia; 15 Department of Community Child Health/Integrated Care, Sydney Children’s Hospital Network, High Street, Randwick, NSW 2031 Australia; 16 Academic Unit of Child Psychiatry, South West Sydney (AUCS), ICAMHS, Mental Health Centre, Liverpool Hospital, Liverpool, NSW Australia; 17 Women and Child Health, Liverpool Hospital, South Western Sydney Local Health District, Liverpool, NSW Australia; 18 Peadiatrics and Neonatology, Liverpool Hospital, South Western Sydney Local Health District, Liverpool, NSW Australia; 19 Maari Ma Health Aboriginal Corporation, Broken Hill, NSW Australia

**Keywords:** Cessation, Exclusive breastfeeding, Australia, Sydney, postnatal period

## Abstract

**Background:**

Optimal breastfeeding has benefits for the mother-infant dyads. This study investigated the prevalence and determinants of cessation of exclusive breastfeeding (EBF) in the early postnatal period in a culturally and linguistically diverse population in Sydney, New South Wales, Australia.

**Methods:**

The study used routinely collected perinatal data on all live births in 2014 (*N* = 17,564) in public health facilities in two Local Health Districts in Sydney, Australia. The prevalence of mother’s breastfeeding intention, skin-to-skin contact, EBF at birth, discharge and early postnatal period (1–4 weeks postnatal) were estimated. Multivariate logistic regression models that adjusted for confounders were conducted to determine association between cessation of EBF in the early postnatal period and socio-demographic, psychosocial and health service factors.

**Results:**

Most mothers intended to breastfeed (92%), practiced skin-to-skin contact (81%), exclusively breastfed  at delivery (90%) and discharge (89%). However, the prevalence of EBF declined (by 27%) at the early postnatal period (62%). Younger mothers (<20 years) and mothers who smoked cigarettes in pregnancy were more likely to cease EBF in the early postnatal period compared to older mothers (20–39 years) and those who reported not smoking cigarettes, respectively [Adjusted Odds Ratio (AOR) =2.7, 95%CI 1.9–3.8, *P* <0.001 and AOR = 2.5, 95%CI 2.1–3.0, *P* <0.001, respectively]. Intimate partner violence, assisted delivery, low socio-economic status, pre-existing maternal health problems and a lack of partner support were also associated with early cessation of EBF in the postnatal period.

**Conclusions:**

Our findings suggest that while most mothers intend to breastfeed, and commence EBF at delivery and at discharge, the maintenance of EBF in the early postnatal period is sub-optimal. This highlights the need for efforts to promote breastfeeding in the wider community along with targeted actions for disadvantaged groups and those identified to be at risk of early cessation of EBF to maximise impact.

## Background

Optimal breastfeeding has both short- and long-term benefits for the mother-infant dyads [1–4]. Breastfed infants are less likely to develop diarrhoea [5, 6], otitis media and obesity as well as have a lower likelihood of mortality [5]. Appropriate breastfeeding is also associated with a higher likelihood of better intellectual functioning [7]. Mothers who engage in optimal breastfeeding practices have a lower risk of developing breast and ovarian cancers, and type 2 diabetes mellitus [4]. The evidence as to why breast milk is the “best” food for the newborn continues to evolve notably. Recent studies have identified mechanisms as to why breast milk is important for the newborn, including stimulation of the immunological and epigenetic functions, and enhancement as well as maintenance of the microbial changes of the gut [8–13]. The World Health Organization and United Nations Children’s Fund (WHO/UNICEF) recommend early initiation of breastfeeding within the first hour of birth and exclusive breastfeeding in the first six months followed by the introduction of safe, age-appropriate and nutritionally adequate complementary foods along with continued breastfeeding until the child is 2 years and beyond [14].

Globally, approximately 38% of infants are exclusively breastfed until around the age of four months, indicating that early cessation of exclusive breastfeeding (EBF) is prevalent in many countries [4]. In Australia, the prevalence of early or timely initiation of breastfeeding is high (96%) [15]. However, EBF for infants aged less than 4 months is low (39%), and decreases even further by 6 months (15%) [13]. The number of Indigenous infants who were exclusively breastfed post discharge from hospital after birth was even lower compared to non-Indigenous infants [16]. Factors that limit optimal breastfeeding in Australian mothers have been elucidated. These included: low socio-economic status (SES) [17], primiparity [18, 19], caesarean delivery, lower maternal age (<25 years) [19], cigarette smoking [20, 21], a mother not having intention to breastfeeding [20–22] and self-reported depressive symptoms [20]. Additionally, anxiety about breastfeeding in public may also be a determinant for suboptimal breastfeeding practices in Australia [23]. A recent study from Western Australia identified potential barriers as breastfeeding problems, poor community acceptability, and inconvenience; while the enablers included breastfeeding education, community support, family support and not having to work [24].

In 2011, the Government of New South Wales (NSW), Australia introduced initiatives and policies (such as policy on promotion, protection and support for breastfeeding and establishment of Breastfeeding Support Clinics) to promote optimal breastfeeding [25–27]. Despite these initiatives, the prevalence of EBF at discharge from health facilities remained unchanged in NSW (80% in 2010 and 79% in 2014) [28], and whether continuing EBF in the early postnatal period has been influenced by these policies is uncertain. We aimed to investigate the prevalence and determinants of cessation of EBF in the early postnatal period in one of Australia’s most culturally and linguistically diverse (CALD) communities in Sydney, NSW, using routinely collected perinatal data. This is the first study in the period post-implementation of breastfeeding initiatives in NSW that examines early cessation of EBF in the postnatal period since the policies were introduced. We will also report on the main reasons why mothers do not initiate or continue EBF in the early postnatal period. It is important to focus on this period as it is considered a critical phase to establish and support appropriate breastfeeding, and when targeted initiatives would yield better outcomes [29–31]. This paper provides breastfeeding information from a high-income country, contrary to a previous study on the importance of breastfeeding, which indicated that researchers and health authorities in developed countries appear to have overlooked breastfeeding [4]. Evidence from this study will provide context-specific information to ensure targeted initiatives on a range of factors underpinning cessation of EBF at the early postnatal period in one of NSW most CALD populations.

## Methods

### Data source

The study used a retrospective clinical audit of all live births in 2014 (*N* = 17,564) in public health facilities in Sydney Local Health District (SLHD) and South Western Sydney Local Health District (SWSLHD). Antenatal data (such as socio-demographic characteristics, history of any previous pregnancy, history of maternal cigarette smoking or alcohol use and mother’s breastfeeding intention) were collected at the first prenatal care visit of mothers by qualified midwives in the local health districts. Similarly, postnatal data (such as information on skin-to-skin contact, EBF at discharge and postnatal depressive symptoms using the Edinburgh Depression Scale) were collected at the post-delivery visit of mothers by qualified nurses, and were stored in the Information Management & Technology Division (IM&TD) database of the local health districts. We obtained these perinatal data with ethics approval, and linked both antenatal and postnatal information using individual identifiers. The New South Wales Perinatal Data Collection – a surveillance system which covers all births in NSW public and private hospitals, and home births – uses similar data to advocate for policy development in the state [28].

### Study setting

SLHD and SWSLHD cover approximately 52% of the Sydney metropolitan region, with a population of 1,457,100 people of different cultural backgrounds [32, 33]. SLHD is located in the centre and inner west of Sydney, and SWSLHD is located in the Sydney south-western region. A number of maternal and child health services are provided to all communities across both districts, including the most socio-economically disadvantaged populations.

### Breastfeeding indicators

The main breastfeeding outcome was EBF in the early postnatal period (i.e., 1–4 weeks postnatal). The prevalence of mother’s breastfeeding intention, skin-to-skin contact, EBF at delivery and discharge stratified by socio-demographic and health service factors were also considered. EBF was based on National Health and Medical Research Council (NHMRC) infant feeding guidelines [34], which are consistent with the WHO/UNICEF definitions for assessing infant and young child feeding practices [35]. For this study, EBF was measured as the proportion of infants who received only breast milk (including expressed milk), but allowed oral rehydration solution, syrups of vitamins/medicines. EBF at delivery was defined as the proportion of infants who received only breast milk in the first 24 h post-delivery, while EBF at discharge was measured as the proportion of infants who received only breast milk, 24 h preceding discharge from the maternity unit. EBF at early postnatal visit referred to the proportion of infants who received only breast milk 24 h prior to the first postnatal health visit. In the local health districts, assessment of both the mother and baby is conducted within 1–4 weeks post-delivery by a community health nurse during a universal home visit, and relevant health information (including breastfeeding data) are entered into the IM&TD database. Data on skin-to-skin contact were collected soon after birth at the maternity unit within the local health districts. Studies have shown that skin-to-skin contact is the most effective approach to promote EBF, regardless of the mode of delivery (vaginal or caesarean births) [36–38].

### Risk factors

For this study, the exposure variables included: maternal age (categorised as <20 years, 20–39 years or ≥40 years), socio-economic status (SES, categorised as high, medium or low), maternal cigarette smoking in pregnancy (categorised as Yes or No), partner support (Yes, Not sure or No), pre-existing maternal health problems (such as diabetes mellitus and/or hypertension, categorised as Yes or No), history of intimate partner violence (IPV, categorised as Yes or No) and type of delivery (categorised as normal vaginal, assisted vaginal or caesarean). SES was based on the Socio-Economic Index for Areas, based on the mother’s address provided, which is a ranking given to areas in Australia according to relative socio-economic advantage and disadvantage, consistent with Australian Bureau of Statistics taxonomy [39]. Deciles of SES were categorised into high (top 10% of the population), middle (middle 80% of the population) and low (bottom 10% of the population) groups as consistent with previous reports [40]. IPV was collected based on the following question: “within the last year, have you been hit, slapped or hurt in other ways by your partner or ex-partner?” Antenatal depressive symptoms were measured using the Edinburgh Postnatal Depression Scale (EPDS, categorised as score ≥13 or score <13) which has been validated and recommended for use in Australia [41]. The CALD aspect of the population on early cessation of EBF was assessed. CALD is a broad and inclusive term for communities with diverse language, ethnic background, nationality, dress, traditions, food, societal structures, art and religion characteristics [42].

### Statistical analysis

Initial analyses involved a series of frequencies and cross-tabulations to determine prevalence of breastfeeding behaviours (i.e., breastfeeding intention, skin-to-skin contact, and EBF at delivery and discharge). Univariate regression models investigated the association between early cessation of EBF and key risk factors, followed by multivariate regression models that incorporated confounders.

The likelihood of a mother ceasing EBF in the early postnatal period and the association with relevant socio-demographic, psychosocial and health service factors was investigated in multivariate logistic regression models. Multivariate models adjusted for the potential confounding factors of maternal body mass index, gender of the baby, maternal alcohol intake and birthing facility location based on previous reports [18, 20].

### Sensitivity analyses

An investigation of the possible effect of missing data on the observed odds ratio was conducted in sensitivity analyses using an imputed dataset based on the original information which comprised of complete outcome data for early cessation of breastfeeding in the early postnatal period. Multivariate imputation by chained equations was used assuming that data were missing at random [43]. This analytical strategy assumes that the known characteristics of study participants can be used to assess the characteristics of individuals with missing data [44]. The outcome and study variables in the main analysis were included in the multiple imputation models. Revised odds ratios from imputed data for comparison with the complete case analysis were generated using the *mim* command. Sensitivity analyses were based on 25 multiple imputations [45], and all analyses were conducted in Stata (Stata Corp, V.14.0, College Station, TX, USA).

### Ethics

Ethics approvals for this study were obtained from the South Western Sydney Local Health District and the Sydney Local Health District Human Research Ethics Committees. Approval numbers HREC: LNR/11/LPOOL/463; SSA: LNRSSA/11/LPOOL/464 and Project No: 11/276 LNR; Protocol No X12-0164 and LNR/12/RPAH/266.

## Results

The study was based on a sample of 17,564 mothers of all live infants born in public facilities in SWSLHD and SLHD. Almost half of the mothers were born outside Australia (46%). Of these, many were from Middle Eastern countries (10%), South East Asia (8%) and Southern Asia (8%), with the cohort covering women from more than 25 countries. Most mothers intended to breastfeed their babies (92%). A large proportion of mothers (81%) practiced skin-to-skin contact (Table 1). EBF at delivery and discharge were high (90 and 89% respectively). In the early postnatal period, more than half of mothers exclusively breastfed their babies (62%), indicating a 27% decrease in EBF prevalence between one and four weeks of delivery. Twenty two percent of mothers provided breast milk and infant formula to their babies, and 16% provided only infant formula. The most common reasons cited for not commencing or continuing EBF in this cohort included: incorrect positioning and attachment, prematurity, low birth weight and jaundice.

**Table 1:** Prevalence of breastfeeding practices by socio-demographic and maternal health characteristics of infant’s mothers from South Western Sydney and Sydney Local Health Districts in 2014 (*N* = 17,564)

	Breastfeeding intention			Skin-to-skin contact			EBF at delivery			EBF at discharge			EBF at 1–4 week		
	*N*	*n*	%	*N*	*n*	%	*N*	n	%	*N*	*n*	%	*N*	*n*	%
SES category	14,752			15,668			14,260			16,073			8459		
High		5487	40.6		5095	40.3		5059	39.5		5791	40.7		1301	15.4
Middle		6190	45.8		5838	46.2		5965	46.6		6479	45.5		4106	48.5
Low		1840	13.6		1703	13.5		1772	13.9		1969	13.9		3052	36.1
Australian born	15,227			16,162			14,698			16,600			8755		
No		6200	44.3		6168	47.3		6129	46.4		6387	43.3		3964	45.3
Yes		7779	55.7		6874	52.7		7084	53.6		8357	56.7		4791	54.7
Maternal age group	15,232			16,169			14,699			16,604			8756		
20–39 years		13,015	93.1		12,129	93.0		12,298	93.1		13,707	92.9		8195	93.6
> 40 years		187	1.3		245	1.9		214	1.6		194	1.3		477	5.5
< 20 years		782	5.6		669	5.1		703	5.3		847	5.7		84	1.0
BMI	14,216			13,382			12,188			13,850			7442		
Underweight		812	6.2		720	6.6		708	6.5		767	6.2		458	6.2
Normal weight		7415	56.9		6282	57.9		6340	57.7		7172	58.1		4512	60.6
Overweight		2900	22.2		2357	21.7		2416	22.0		2704	21.9		1606	21.6
Obese		1915	14.7		1489	13.7		1517	13.8		1702	13.8		866	11.6
Pre-existing maternal health problems	13,838			14,976			13,550			15,250			8122		
No		8815	69.4		9076	74.5		8872	72.9		9777	72.2		5898	72.6
Yes		3886	30.6		3103	25.5		3297	27.1		3759	27.8		2224	27.4
Intimate partner violence	13,972			13,136			11,964			13,576			7280		
No		12,634	98.6		10,526	98.6		10,628	98.8		11,932	98.8		7200	98.9
Yes		175	1.4		147	1.4		134	1.3		146	1.2		80	1.1
Type of delivery	15,195			16,127			14,660			16,565			8732		
Normal vaginal		8720	62.5		10,017	77.0		9111	69.2		9301	63.2		5640	64.6
Assisted vaginal		1516	10.9		1472	11.3		1450	11.0		1604	10.9		991	11.4
Caesarean section		3711	26.6		1514	11.6		2615	19.9		3806	25.9		2101	24.0
Alcohol consumption	14,606			13,795			12,567			14,292			7668		
No		13,186	98.5		11,026	98.3		11,160	98.5		12,549	98.4		7537	98.3
Yes		205	1.5		189	1.7		174	1.5		203	1.6		131	1.7
Smoking status	15,226			14,538			13,239			15,097			8085		
No		13,104	93.7		10,948	92.5		11,212	93.6		12,727	94.2		7769	96.1
Yes		874	6.3		892	7.5		764	6.4		781	5.8		316	3.9
Supportive partner	13,952			13,104			11,941			13,550			7316		
Yes		12,444	97.0		10,308	96.8		10,464	97.1		11,735	97.1		7132	97.5
Not sure		126	1.0		107	1.0		99	0.9		122	1.0		55	0.7
No		252	2.0		230	2.2		209	1.9		235	1.9		129	1.8
Antenatal depressive symptoms	13,368			12,549			11,450			12,963			6976		
EPDS <13		11,524	94.0		9586	94.3		9702	94.3		10,840	94.1		6581	94.4
EPDS ≥13		732	6.0		576	5.7		586	5.7		682	5.9		395	5.6

*N* Sample size, *n* positive cases; pre-existing maternal health problems included diabetes mellitus and/or hypertension

EBF at delivery was defined as infants who received only breast milk within the first 24 h post-delivery

EBF at discharge was measured as infants who received only breast milk in the 24 h preceding discharge from the maternity unit

Younger mothers (<20 years) were significantly more likely to discontinue EBF in the early postnatal period compared to older mothers (20–39 years) [Adjusted Odds Ratio (AOR) =2.7, 95%CI 1.9–3.8, *P* <0.001] (Table 2). Mothers from higher SES groups were significantly less likely to cease EBF in the early postnatal period compared to those from lower SES groups (AOR = 0.6, 95%CI 0.5–0.9, *P* <0.001). Mothers who reported smoking cigarettes in pregnancy were significantly more likely to stop EBF in the early postnatal period compared to their counterparts (AOR = 2.5, 95%CI 2.1–3.0, *P* = 0.042). The odds for ceasing EBF in the early postnatal period were higher among mothers who received interventions during delivery (AOR = 1.5, 95%CI 1.4–1.7, *P* <0.001 for caesarean section and AOR = 1.3, 95%CI 1.1–1.5, *P* <0.001 for assisted vaginal delivery) and, those who reported a history of intimate partner violence (AOR = 1.4, 95%CI 1.0–2.0, *P* = 0.042). Mothers who reported not having partner support were significantly more likely to discontinue EBF in the early postnatal period compared to their counterparts (AOR = 1.7, 95%CI 1.2–2.1, *P* = 0.003). The analysis showed no association between CALD population and early cessation of EBF in the postnatal period. Findings from multiple imputation analyses were not substantially different from the complete case analysis for most of the study factors (Table 2), suggesting that missing data did not considerably affect the observed findings.

**Table 2:** Associations between key study factors and early cessation of exclusive breastfeeding in the postnatal period of infant’s mothers from South Western Sydney and Sydney Local Health Districts in 2014 (*N* = 17,564)

	Complete case analysis				Multiple imputation analysis^a^			
	Unadjusted OR (95%CI)	*P* value	Adjusted OR (95%CI)^b^	*P* value	Unadjusted OR (95%CI)	*P* value	Adjusted OR (95%CI)^b^	*P* value
Early cessation of EBF in early postnatal period								
Maternal age group								
20–39 years	1.0		1.0		1.0		1.0	
> 40 years	1.1 (0.9–1.3)	0.293	1.1 (0.9–1.4)	0.236	1.1 (0.9–1.3)	0.524	0.9 (0.7–1.2)	0.225
< 20 years	3.1 (2.4–4.1)	<0.001	2.7 (1.9–3.8)	<0.001	3.4 (2.4–4.7)	<0.001	2.9 (2.1–4.1)	<0.001
Socio-economic status								
Low	1.0		1.0		1.00		1.0	
Medium	0.8 (0.6–0.7)	<0.001	0.7 (0.6–0.8)	<0.001	0.7 (0.6–0.7)	<0.001	0.7 (0.6–0.7)	<0.001
High	0.5 (0.4–0.6)	<0.001	0.6 (0.5–0.9)	<0.001	0.5 (0.4–0.6)	<0.001	0.5 (0.5–0.6)	<0.001
Antenatal depressive symptoms								
EPDS <13	1.0		1.0		1.00		1.0	
EPDS ≥13	1.2 (1.0–1.4)	0.014	1.2 (1.1–1.5)	0.068	1.2 (1.1–1.2)	0.016	1.3 (1.1–1.5)	0.056
Cigarette smoking								
No	1.0		1.0		1.0		1.0	
Yes	3.2 (2.8–3.7)	<0.001	2.5 (2.1–3.0)	<0.001	2.9 (2.5–3.5)	<0.001	2.7 (2.3–3.2)	<0.001
Supportive Partner								
Yes	1.0		1.0		1.0		1.00	
Unsure	1.9 (1.3–2.8)	<0.001	1.6 (1.1–2.4)	0.026	1.8 (1.2–2.7)	0.007	1.5 (1.0–2.3)	0.078
No	1.5 (1.2–2.0)	<0.001	1.7 (1.2–2.1)	0.003	1.7 (1.3–2.3)	<0.001	1.4 (1.3–1.9)	0.049
CALD								
No	1.0							
Yes	0.9 (0.8–1.0)	0.107	0.9 (0.8–1.0)	0.088	1.1 (0.9–1.2)	0.0101	0.9 (0.7–1.0)	0.075
Pre-existing maternal health problems								
No	1.0		1.0		1.0		1.0	
Yes	1.1 (1.0.–1.2)	0.207	1.2 (1.1–1.3)	0.002	1.1 (1.1–1.2)	0.095	1.0 (0.9–1.1)	0.405
Intimate partner violence								
No	1.0		1.0		1.0		1.0	
Yes	1.6 (1.2–2.2)	0.004	1.4 (1.0–2.0)	0.042	1.7 (1.2–2.5)	0.005	1.2 (0.7–1.8)	0.516
Type of delivery								
Normal vaginal	1.0							
Assisted vaginal	1.1 (1.0–1.2)	0.119	1.3 (1.1–1.5)	<0.001	1.0 (0.9–1.1)	0.898	1.1 (0.9–1.2)	0.426
Caesarean section	1.5 (1.4–1.6)	<0.001	1.5 (1.4–1.7)	<0.001	1.6 (1.3–1.6)	<0.001	1.5 (1.3–1.6)	<0.001

^a^Sensitivity analyses following multiple imputations for missing values; *CALD* culturally and linguistically diverse population; pre-existing maternal health problems included diabetes mellitus and/or hypertension

^b^Adjusted for maternal body mass index, gender of the baby, maternal alcohol intake and birthing facility location. *N* Sample size, *n* positive cases

## Discussion

In this study, most mothers intended to breastfeed, and most infants experienced skin-to-skin contact. EBF prevalence at delivery and at discharge from hospital after birth were high, but the prevalence of EBF in the early postnatal period declined by 27%, suggesting that many infants were introduced to complementary foods as early as the second month of birth, contrary to recommended practice. Younger mothers (<20 years) and those with no supportive partners were significantly more likely to discontinue EBF compared to older mothers (20–39 years) and those who reported having supportive partners, respectively. Mothers from higher SES groups were less likely to cease EBF compared to those from low SES groups. The odds for ceasing EBF in the early postnatal period were higher among mothers who received interventions during delivery, pregnant mothers who smoked cigarettes in pregnancy, and those who reported a history of intimate partner violence (IPV). The negative impact of antenatal depressive symptoms on cessation of EBF in this population has been reported elsewhere [40].

EBF decreased in the early postnatal period, consistent with previous reports [15, 21]. Previous Australian studies revealed that this steep decline in EBF may be due to early return to work [46], inexperience in breastfeeding, a lack of partner’s support [15], and Aboriginal heritage [21]. Additionally, our study found that younger maternal age and smoking in pregnancy were the strongest determinants (in terms of effect sizes) for sub-optimal EBF in Sydney. This finding is consistent with a previous Australian study, which indicated that Indigenous status, young maternal age and smoking in pregnancy were associated with sub-optimal breastfeeding [21]. Current interventions to promote breastfeeding in NSW include: policy on promotion, protection and support for breastfeeding [27]; maternity leave policy; establishment of Breastfeeding Support Clinics; Breastfeeding Reference Groups [26, 32, 47]; and a universal home visiting program [48, 49]. Some aspects of these interventions specifically target vulnerable and at-risk mothers (such as low SES mothers) who may have limited information in appropriate breastfeeding [49]. Our study provides further insight into challenges in maintaining EBF in the early postnatal period, and suggests that current and/or future interventions to promote EBF should target disadvantaged groups to maximise positive results.

Consistent with prior studies [17, 19], we found that higher maternal SES was associated with EBF compared to mothers from lower SES groups, suggesting better uptake of health information in higher SES mothers. Mothers with higher educational attainment are more likely to be in employment as well as have a better propensity to take up health care messages compared to those with lower educational attainment [50, 51]. Other plausible reasons for why mothers from lower SES groups engaged in suboptimal breastfeeding practices may include limited skills to negotiate working hours, stress and poor social interactions [52, 53]. These discrepancies in breastfeeding practices across the socio-economic scale may also reflect aspects of health inequalities between higher SES and lower SES families in Australia [51]. Legislators, social and health administrators must work together to improve infant feeding behaviours in all households to ensure improvement in household well-being and productivity.

The effective implementation of the Baby Friendly Hospital Initiative (BFHI) is an important strategy to increase optimal breastfeeding rates following caesarean delivery. BFHI was introduced in 1990 by WHO/UNICEF, and was implemented in Australia in 1993 to promote, protect and support breastfeeding in the hospital and community [54]. Among Australian states and territories, and in comparison to other states such as Queensland (28%) and South Australia (18%), NSW has one of the lowest proportions of BFHI accredited facilities (14%) [54]. This is particularly significant considering the fact that NSW is the most populous state in Australia [55]. The current policy direction for breastfeeding promotion, protection and support in the state includes important priority areas for action such as health professionals’ education and training; support for breastfeeding in health care settings; breastfeeding support for priority groups; and continuity of care, referral pathways and support networks [27]. Appropriate implementation and sustained monitoring of these key areas of optimal breastfeeding across different levels of cultural background, socio-economic and demographic measure is crucial to promote EBF in Sydney, Australia.

In Australia and internationally, previous studies [56–61] have shown that family members (particularly partner or grandmother) do not only influence the decision to initiate and continue EBF, but they can also play a role  in premature cessation of EBF. Our study indicated that mothers with no supportive partners were more likely to discontinue EBF in the early postnatal period. Evidence indicates that fathers want to help the mother to have a successful breastfeeding experience [58, 62]. However, limited breastfeeding information for fathers and conflicting information from health professionals to fathers were reported as barriers to fathers’ participation in breastfeeding support. Providing fathers with appropriate breastfeeding information to become breastfeeding advocates will increase EBF duration in Sydney [63]. Appropriate implementation of the BFHI will also address aspects of the inconsistency in breastfeeding information from health professionals. Our study also indicated that IPV and maternal cigarette smoking were associated with cessation of EBF in the early postnatal period. The Australian and NSW Government initiatives to stop IPV in communities [64] and control tobacco smoking [65] are strategies needed at the national and sub-national level to improve breastfeeding practices of mothers. These efforts, however, must be tailored to the socio-economic environment in which mothers raise their children to ensure better outcomes.

In comparison to many developing countries [6, 66–68], the introduction of solid, semi-solid and soft foods to infants aged less than 6 months may not create the environment for Australian infants to experience diarrhoea associated with inappropriate infant feeding practices. Reasons for this observation may include better social amenities (like housing, access to potable water and good sanitary environment), and better availability and affordability of food storage systems, operational policies and vaccination programs in Australia. Although infants in Australia may have a lower likelihood of developing diarrhoea associated with suboptimal infant feeding, appropriate breastfeeding remains relevant in a developed country, such as Australia. For example, breastfeeding is relevant in reducing the risk for a number of non-communicable diseases in mother-infant pairs, including obesity [69] diabetes [70] and cancers [71–73] in addition to facilitating optimal development and cognitive abilities for infants [7]. Similarly, a recent randomised control trial among Australian children showed that optimal breastfeeding was associated with a reduced risk of childhood obesity [74, 75]. Initiatives to improve mother and infant health in NSW must consider the important role of optimal breastfeeding to maximise outcomes.

An emerging threat to optimal breastfeeding in the 21^st^ century may be the continued “disapproval about breastfeeding in public”. In Australia, the law supports nursing mothers to breastfeed in public places [76]. However, shop attendants and security guards [77–80] in Australia have asked mothers to leave a public venue whilst breastfeeding, suggesting that a proportion of people are unaware of the mother’s right to breastfeed. Although our study did not examine the impact of disapproval about public breastfeeding, previous studies have indicated that anxiety associated with public breastfeeding was a major reason for suboptimal breastfeeding [81–83]. Even though responses to public breastfeeding can be positive or negative in the community [77, 84], interventions to promote breastfeeding in the wider community must not only focus on the importance of breastfeeding, but also on normalising public breastfeeding. Research into strategies to make public breastfeeding an acceptable norm in the community may also be warranted.

### Study limitations and strengths

The study has a number of limitations. First, the outcome was measured based on self-report which may have led to a recall and/or measurement bias that may have underestimated or overestimated the association between early cessation of EBF and key study factors. Second, unmeasured confounding factors (such as culture, multi-parity or level of support services received postnatal) may also affect the study findings. Third, longitudinal data on EBF (from 4 weeks to 6 months postnatal) were unavailable, information that may have provided a broader pattern of EBF of mothers in Sydney. Finally, our analysis was unable to separate mothers who were assessed by the community health nurse in the first, second, third or fourth week postnatal. This information would have provided additional detail on early cessation of EBF in the postnatal period. Despite these limitations, the study provides information on EBF in the early period following delivery in one of the most diverse populations in Australia. We believe that any potential bias due to missing data is unlikely to have affected the observed findings since the study took this into consideration using a sensitivity analysis that imputed missing data. Our study also provides breastfeeding information from a high-income country, contrary to a proposition from a previous study, which indicated that researchers and health jurisdictions in high-income countries appear to have ignored breastfeeding [4].

## Conclusions

Our findings suggest that many mothers intended to breastfeed, and most mothers practiced skin-to-skin contact, EBF at delivery and at discharge from hospital after birth. However, the prevalence of EBF declined in the early postnatal period. Younger mothers (<20 years), having an unsupportive partner, intimate partner violence, and interventions during delivery were associated with early cessation of EBF at the postnatal period in Sydney, Australia. Our study provides further insight into breastfeeding practices in one of Australia’s most culturally and linguistically diverse populations to ensure targeted initiatives for all mothers, especially those identified to be at risk of early cessation of EBF. Initiatives that incorporate wider consultative processes will maximise impact and long-term sustainability.

## Abbreviations

CALD: Cultural and Linguistically Diverse
EBF: Exclusive breastfeeding
SLHD: Sydney Local Health District
SWLHD: South Western Sydney Local Health District
UNICEF: United Nations Children’s Fund
WHO: World Health Organization
